# Step-by-Step Implementation of Three-Dimensional Print Technology in Preoperative Neurosurgery Planning

**DOI:** 10.7759/cureus.67119

**Published:** 2024-08-18

**Authors:** Todor G Bogdanov, Rene Mileva, Dilyan Ferdinandov

**Affiliations:** 1 Department of Medical Physics, Medical University – Sofia, Sofia, BGR; 2 Department of Physiology, Faculty of Medicine, Medical University – Sofia, Sofia, BGR; 3 Department of Neurosurgery, Faculty of Medicine, Medical University – Sofia, Sofia, BGR; 4 Clinic of Neurosurgery, St. Ivan Rilski University Hospital, Sofia, BGR

**Keywords:** tumor segmentation, medical imaging, open-source software, preoperative planning, neurosurgery, 3d printing

## Abstract

This study presents a detailed methodology for integrating three-dimensional (3D) printing technology into preoperative planning in neurosurgery. The increasing capabilities of 3D printing over the last decade have made it a valuable tool in medical fields such as orthopedics and dental practices. Neurosurgery can similarly benefit from these advancements, though the creation of accurate 3D models poses a significant challenge due to the technical expertise required and the cost of specialized software. This paper demonstrates a step-by-step process for developing a 3D physical model for preoperative planning using free, open-source software. A case involving a 62-year-old male with a large infiltrating tumor in the sacrum, originating from renal cell carcinoma, is used to illustrate the method. The process begins with the acquisition of a CT scan, followed by image reconstruction using InVesalius 3, an open-source software. The resulting 3D model is then processed in Autodesk Meshmixer (Autodesk, Inc., San Francisco, CA), where individual anatomical structures are segmented and prepared for printing. The model is printed using the Bambu Lab X1 Carbon 3D printer (Bambu Lab, Austin, TX), allowing for multicolor differentiation of structures such as bones, tumors, and blood vessels. The study highlights the practical aspects of model creation, including artifact removal, surface separation, and optimization for print volume. It discusses the advantages of multicolor printing for visual clarity in surgical planning and compares it with monochromatic and segmented printing approaches. The findings underscore the potential of 3D printing to enhance surgical precision and planning, providing a replicable protocol that leverages accessible technology. This work supports the broader adoption of 3D printing in neurosurgery, emphasizing the importance of collaboration between medical and engineering professionals to maximize the utility of these models in clinical practice.

## Introduction

In today's world, precise planning of an operation and minimizing its execution time are of utmost importance. The development of technologies for three-dimensional (3D) printing of physical objects over the past 10 years offers increasing possibilities for assisting in this process. In the fields of orthopedics and dental practice, the use of these technologies for creating models for preoperative planning or surgical guidance is approaching the status of a gold standard. Potential applications are also emerging in various other fields, for example, in dental medicine [1-3] and orthopedics [4-6]. Neurosurgery is no exception [7-9] and can also benefit from this technology for the precise study of a specific case with its anatomical peculiarities. However, there is a significant barrier to this - the necessity of creating the model itself, which is generally a non-trivial and time-consuming task. It requires familiarity with numerous software programs and training to work with them. Many of these programs are under paid licenses, and due to their specialized nature, these licenses can cost thousands of euros annually. This publication aims to present a step-by-step process for creating a 3D physical model for preoperative planning using open-licensed software, illustrating this with a real medical case of metastases originating from renal cell carcinoma. This will significantly contribute to the medical community, enabling any team to reproduce the study step by step for their specific case.

## Technical report

This study presents results obtained from processing a computed tomography (CT) scan of a patient using a 16-multidetector CT scanner Tomoscan AV (Philips, Amsterdam, Netherlands). The creation of the initial 3D object from the DICOM file dataset was carried out using InVesalius 3 [10,11], an open-source software for the reconstruction of CT and magnetic resonance images. The focus on the software for this research is not only due to its open-source nature but also because of its availability across different platforms such as Microsoft Windows, GNU/Linux, and Apple Mac OS X. After creating the initial 3D structure. It was exported in STL file format and further processed and prepared for printing.

The subsequent processing was done using Autodesk Meshmixer 3.5 (Autodesk, Inc., San Francisco, CA), which operates under Microsoft Windows, but Blender 4.1.1, which runs on various operating systems, can also be used. After the necessary processing, the object was exported in STL file format and prepared for printing.

The 3D object was printed using a Bambu Lab X1 Carbon 3D Printer (Bambu Lab, Austin, TX) with the combo option for multicolor printing. This allows the created object, including the bone structure of the tailbone and spine, carcinoma, and vascular system in the area of interest, to be printed simultaneously in different colors for differentiation. Monochromatic printing or segmented printing of individual structures is also an option and will be considered from the process optimization perspective.

Large infiltrating tumors involving the sacrum are complex challenges in surgical planning and intervention. In this context, we present a case of a 62-year-old male patient with arterial hypertension and no significant comorbidities, who sought medical attention due to progressively worsening pelvic and bilateral lower limb pain over two months. Concurrently, the patient experienced leg edema while maintaining unaffected muscle strength and urinary control.

The imaging studies, including pelvic and lumbar spine MRI and whole-body CT, revealed a substantial infiltrating tumor extensively engaging the sacrum. This tumor exerted compression on the lumbosacral plexuses and bilateral pelvic vessels, explaining the patient's symptoms. The preoperative angiography further highlighted the intricate vascular supply originating from both iliac and rectal arteries, necessitating partial embolization to mitigate intraoperative bleeding.

The obtained medical image from the CT scanner in the arterial phase consists of 424 slices with a resolution of 1.50 mm. After importing it into InVesalius 3.1, a Hounsfield unit range of 100 to 2296 was selected. It is important to note that the software provides predefined ranges for bones (compact and spongy), soft tissues, muscles, and others, but manually expanding the range is recommended to ensure coverage in the area of interest.

**Figure 1:**
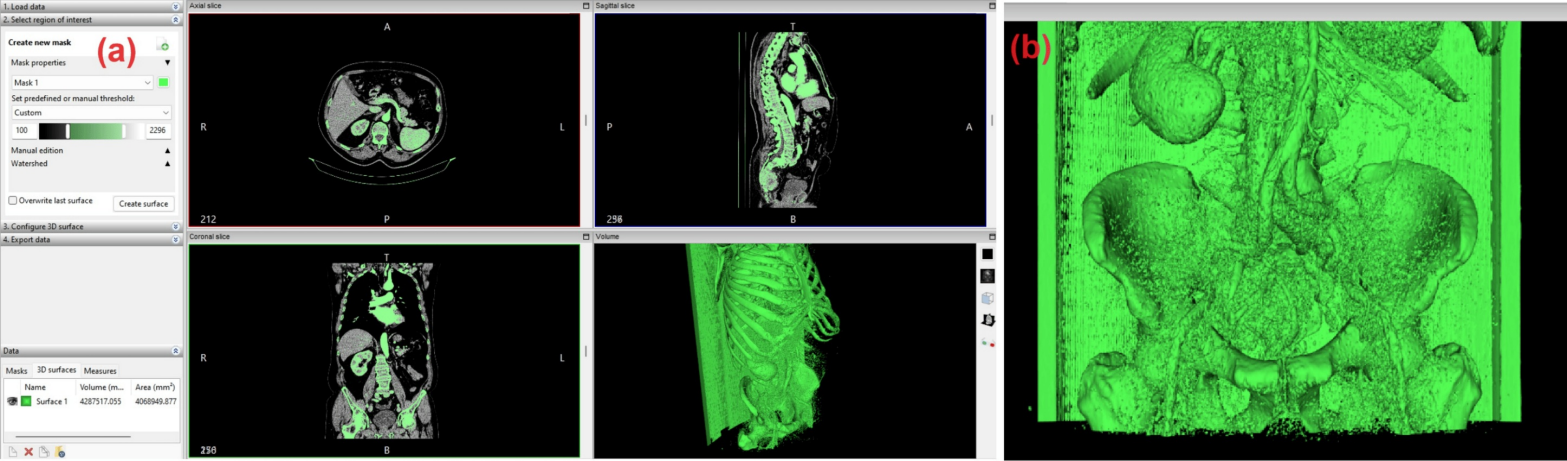
Reconstructed surface by InVesalius software.

The visualization of the reconstructed object is presented in Figure 1. As seen in Figure 1A, there is a cloud of objects in the vicinity of the area of interest, along with the visualization of the CT scanner mass. It is possible to select the area of interest and/or optimize the volume of the generated object, but the necessity for subsequent processing to prepare the file for printing makes this time-consuming task unnecessary.

The created object is exported as a 3D object in STL format for further processing. If the 3D visualization on the software screen does not satisfy the researchers, an alternative one can be created by selecting a different region of interest. However, we recommend not replacing the current one due to the need for comparison. It is highly advisable to save the progress of the current visualization to minimize the time required for re-visualization.

The created and exported file is imported into Autodesk Meshmixer software for processing. It is important to note that processing in these programs is a kind of art interpretation and may lead to a distortion of accuracy. Therefore, the authors do not recommend deviating from the set procedure. In this step of preparing the file for printing, a series of steps are performed aiming at (1) specifying the area of interest; (2) removing artifacts from the CT scan; (3) separating the individual anatomical structures in the area of interest; (4) reducing the volume for printing; and (5) creating an STL file or files to be directed to slicer software.

**Figure 2:**
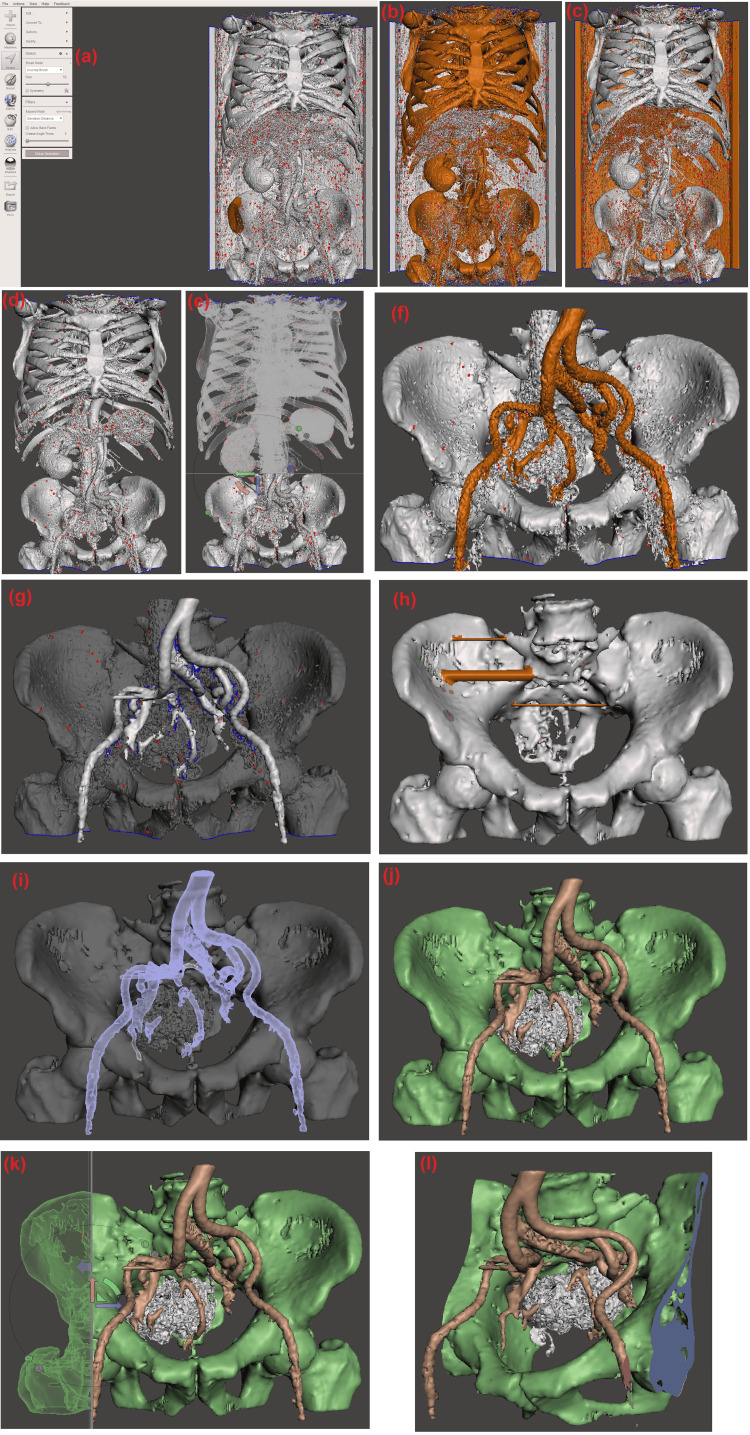
Autodesk Meshmixer - evolution of the steps.

Figure 2 presents the individual stages through which processing in Autodesk Meshmixer passes.

The imported file contains numerous artifacts in a cloud-like formation that are not connected to the main anatomical structure. The first task is to remove this cloud by selecting and deleting these elements. Selecting the individual elements is not a trivial task since they are not connected, but there is an elegant solution. Using the selection tool, we will mark the bone structure in the pelvic area. From the “Select” menu, choose “Unwrap Brush” with a size of around 50 units. Using the pointer of the computer mouse, mark a segment of the main anatomical structure, as shown in Figure 2A. By double-clicking the left mouse button on the marked section or selecting “Select >> Modify… >> Expand to connected” from the left menu, the entire surface connected to the marked section will be selected. The result is shown in Figure 2B. This is the structure of interest to us. Therefore, it is necessary to select all unselected elements for deletion. From the menu, choose “Select >> Modify… >> Invert.” The result (Figure 2C) is an inverted selection. Using the “Delete” button on the keyboard, delete the selected elements to obtain the primary segmented anatomical image shown in Figure 2D. As visible, the area of interest is part of the segmented structure. This area is cut out to optimize the workflow. A section of the structure is cut using the “Edit >> Plane cut” menu. It is recommended to maintain the object's orientation in space throughout the steps performed so far. This can be achieved by consistently selecting the same plane each time from the spatial orientation controller in the upper right corner. The plane's position can be adjusted according to the requirements of the work volume. Its positioning can be done using the navigational coordinate system (visible in Figure 2E) in the plane's center along which the cut will be made. This can also be done by marking the orientation in the projection plane using the right mouse button to mark the start and end points.

After making the cut, the structure is reduced, but it is still not ready for export and printing. In this particular case, we have a pelvic bone with a carcinoma. The arterial blood vessels are nearby and even in contact with the formation. For the accurate creation of the model for preoperative planning, these elements should be separated from each other. Using the “Select” function with the “Sphere Brush,” mark the outermost structure. By using the brush and following the surface of one anatomical structure, we can observe that a partial selection of the contact area between the different structures occurs. This is how the plane of separation is determined. In our specific case, this is the vascular system. After marking it, as shown in Figure 2F, this surface should be separated from the others. Using the left menu, after marking the desired surface, choose “Select >> Edit >> Separate.” This will separate the surface from the others and create an "Object" list where it will be an individual object, as shown in Figure 2G. From the "View" menu, you can hide or display the object list.

We repeat the procedure for separating the surface of the tumor from that of the bone structure. Separating tumor tissue from bone structure is challenging due to their integration. Complete separation of these tissues is practically impossible, and therefore, the precision of marking and the boundary of separation depend on the operator's interpretation. This is also true for the surgeon during an operation, where the accuracy of recognizing and separating tumor tissue is similarly reliant on the specialist's experience and skills. The result should be the presence of three 3D objects in the object list. By hiding all of them and visualizing them one by one using the option to the right of each one in the list, it becomes apparent that these are not 3D objects in the sense of volume. The objects represent bent surfaces and, in most cases, are not connected or have discontinuities. Our approach is to turn these bent planes in space into solid objects, as shown in Figure 2H.

To do this, selecting an object from the list and transforming it into a solid using the “Edit >> Make Solid” menu is necessary. It is recommended to choose “Accurate” for the “Solid type.” By adjusting all other parameters and using the “Update” option, the solid object to be obtained can be visualized, and upon selecting “Accept,” the transformation is confirmed. This should be repeated for each of the previously separated surfaces.

Examining Figure 2H, we notice atypical elements in the created 3D solid object. These are often due to opposite gaps in the surface. These structures should be marked and deleted using the “Delete” button on the keyboard, resulting in “holes” in the object’s surface. Using the “Select” tool, we can mark the contour and use the “Edit >> Flat Fill” command to close these openings in the surface.

The created surfaces are now separated from each other and can be visualized individually, as seen in Figure 2I. The resulting set of surfaces should be combined into one structure consisting of separate surfaces while maintaining their absolute coordinates. For accurate preoperative planning, it is highly recommended not to move the objects in space relative to each other. When planning the area of interest, the technical capabilities of the chosen printing equipment should also be considered. A primary limiting parameter is the print volume. In our case, this is 256 x 256 x 256 mm, necessitating either shrinking the area of interest or scaling the object.

Figure 2K shows an approach where the detail volume is reduced using a plane cut. From the “Edit >> Plane Cut” menu, a plane cut is specified and positioned in the global coordinate system. Using this tool, we achieve a reduction in the print volume and obtain the final detail visible in Figure 2L.

The prepared visualization case is exported as a volumetric file with an STL extension using the “Export” option. The resulting file is ready for printing as a single combined object. The complexity of the objects within it subsequently leads to time constraints for the production of the detail. Our chosen printer, the Bambu Lab X1 Carbon Combo, offers the capability for multicolor printing, which is performed sequentially in each layer of the visualization model after slicing the detail in the proprietary Bambu Studio software (Bambu Lab).

**Figure 3:**
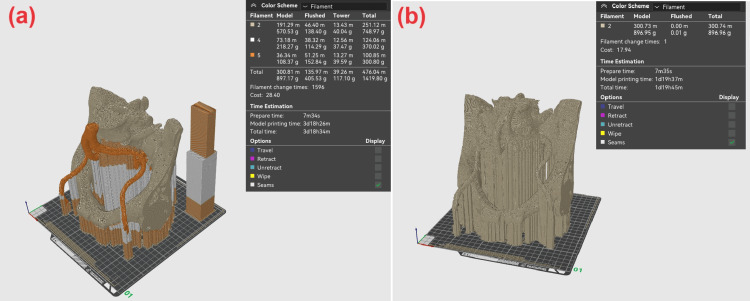
Sliced multi-surface structure in multicolor (a) and single-color (b) mode of printing.

In the example we examined, we have three surfaces (bones, vascular system, and tumor); meaning the option for three separate colors for printing can be selected. As seen in Figure 3A, the time required for such a print is 90 hours and 34 minutes. For single-color printing with polylactic acid (PLA) material (Figure 3B), the print time is approximately 43 hours and 45 minutes, which is half of the multicolor print time. This is due to the time needed for the machine to perform 1,596 material changes to individualize the color of the separate objects. Notably, the multicolor print consumes about 520 g of material out of the total 1,420.80 g needed for color changes, or nearly 37% of the material.

**Figure 4:**
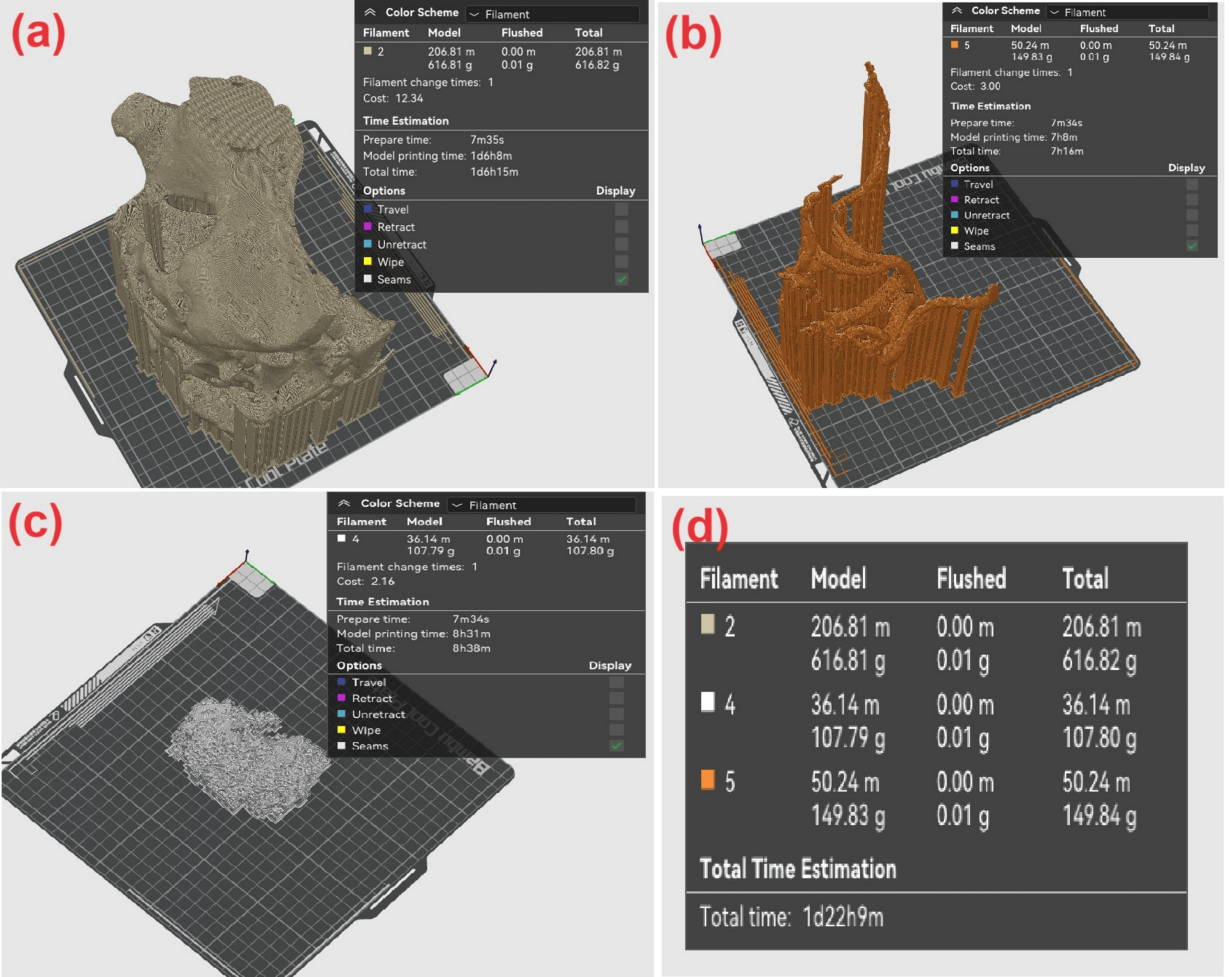
Sliced structures in case of one-by-one structure printing.

We also consider an alternative approach for creating a multicolor print, where each structure in the examined visualization volume is exported into a separate STL file and printed individually in the chosen color. Figure 4 shows visualizations from the Bambu Studio program for each of the exported models: Figure 4A for the bone structure, Figure 4B for the blood vessels, and Figure 4C for the tumor.

As shown in Figure 4D, the total print time is comparable to the time required for printing a monochrome detail, with a difference of just under six hours in favor of monochrome printing. However, if at least two printers are available, the time will be reduced to the duration needed to print the largest detail, which is the bone structure with a print time of 30 hours and 15 minutes.

**Figure 5:**
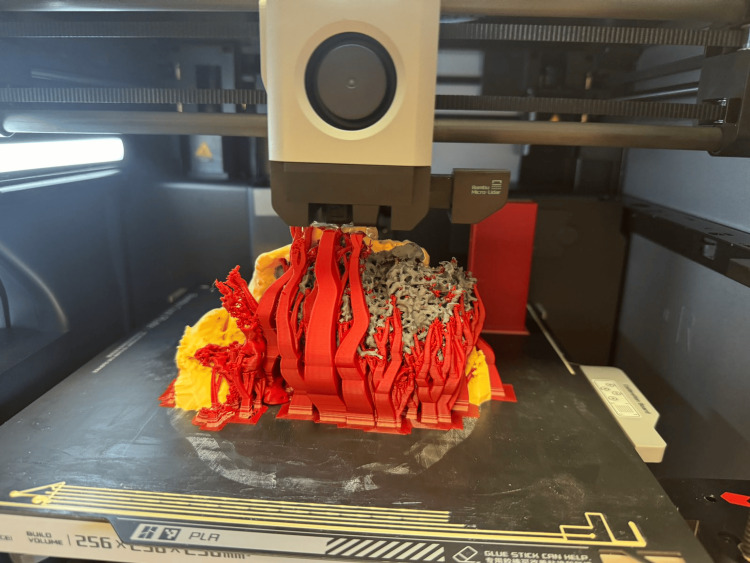
3D printed detail during the process of multi-color printing.

Figure 5 presents the printed detail used for preoperative planning in the examined case. It was printed as a single multicolor object.

It should be noted that changing the print time can also be achieved by adjusting settings for support structures, altering slice thickness, and/or the number of walls along with the fill density of the model during printing. However, this does not necessarily lead to real-time optimization due to the potential risk of defects during printing. Such defects, though not inevitable, may require restarting the printing process from the beginning, resulting in a loss of achieved progress.

## Discussion

The integration of 3D printing technology into preoperative neurosurgery planning presents a transformative advancement in surgical precision and patient outcomes. This study demonstrates a viable and cost-effective method for generating accurate 3D models using open-source software, addressing several critical challenges faced by neurosurgeons. The cost of a printed detail depends on the price of the material used. PLA filament, for example, can be purchased for between 12 and 100 euros. Considering machine depreciation, labor hours, and electricity consumption, the cost of producing details can vary significantly. For our example, the cost of the detail does not exceed 10 to 15 euros, taking into account all the specific aspects of the process involved in its creation.

One of the most significant advantages of using 3D printed models is the enhanced visualization of complex anatomical structures. Traditional imaging techniques, such as CT and MRI scans, provide valuable data but often require substantial expertise to interpret effectively. The physical 3D models allow surgeons to interact with patient-specific anatomy in a tangible way, improving their spatial understanding and facilitating more precise surgical planning. This tactile interaction is particularly beneficial in complex cases, such as the sacral tumor discussed in this study, where precise delineation of tumor margins and critical structures is crucial.

Our methodology emphasizes accessibility and affordability, utilizing free software tools such as InVesalius 3 and Autodesk Meshmixer. This approach democratizes advanced surgical planning techniques, making them available to institutions with limited resources. The successful application of this method in a clinical case underscores its practicality and effectiveness. By providing detailed steps, we aim to empower other medical teams to adopt and adapt this technology to their specific needs.

The use of multicolor printing is another innovative aspect highlighted in this study. By differentiating anatomical structures through color, the models provide clearer visual cues, aiding in the identification of critical areas such as blood vessels and tumor boundaries. This level of detail can significantly impact surgical strategy, potentially reducing operative time and improving patient safety. Comparatively, monochromatic models, while still useful, may not offer the same level of immediate clarity, emphasizing the added value of multicolor capabilities.

However, the implementation of 3D printing in neurosurgery is not without challenges. The process of image segmentation and model optimization requires technical expertise and can be time-consuming. Additionally, ensuring the accuracy of the printed models is paramount, as any discrepancies can lead to suboptimal surgical outcomes. Future advancements in software automation and printer technology are likely to mitigate these issues, making the process more efficient and user-friendly.

Despite these challenges, the benefits of 3D printing in preoperative planning are compelling. Enhanced visualization improved surgical accuracy, and the potential for better patient outcomes make it a valuable tool in the neurosurgical arsenal. Our study contributes to the growing body of evidence supporting 3D printing in medicine and provides a practical framework for its implementation.

The integration of 3D printing into preoperative planning for neurosurgery is an area of growing interest, supported by numerous studies. Recently, several studies have shown that the use of 3D models significantly improves surgical accuracy and reduces operative time [12,13]. Our research confirms these findings by presenting a specific case where the use of a 3D model enhanced visualization and preparation for complex surgery.

Furthermore, the use of open-source software and relatively affordable printing technology makes this methodology cost-effective and accessible to a wide audience. This is consistent with previous studies [14,15], which emphasize the importance of the accessibility of medical technologies for improving healthcare globally.

However, our study has some limitations that need to be addressed in future research. For instance, as noted by Fogarasi et al. [16], the accuracy of 3D models can vary depending on the quality of the source images and the software platform used. We also observed that the process of segmentation and optimization requires significant technical skills, which may be a barrier to its widespread application. Despite the promising results and potential of the developed methodology, the study has several limitations. First, the process of segmentation and model optimization requires significant technical skills and time, which may limit its widespread application in clinical practice, especially in institutions with limited staff and resources. Second, the accuracy of the printed models depends on the quality of the initial images from the CT or MRI scanners, as well as on the settings of the software and printer. There may be slight discrepancies between the digital models and the physical prints, which could affect surgical precision. Third, the study focuses on a single clinical case, which limits the ability to generalize the results to a broader range of cases and anatomical variations. Additional research with a larger number of patients and different types of tumors is necessary to confirm and extend the conclusions. Finally, while the open-source software tools used are accessible, they may have limitations in terms of functionality and support, which could necessitate the use of commercial solutions for more complex cases.

Future studies should address these limitations to refine and validate the methodology, expand its scope, and improve the accuracy and efficiency of 3D models for preoperative planning in neurosurgery. In conclusion, the step-by-step methodology presented in this study offers a robust and accessible means of integrating 3D printing into neurosurgery. As technology continues to evolve, adopting such innovative tools will likely become more widespread, ultimately enhancing the quality of care provided to patients.

## Conclusions

The present study demonstrates the significant potential of 3D printing technologies to enhance preoperative planning in neurosurgery. By developing a cost-effective and accessible methodology that utilizes open-source software and relatively affordable printing technologies, we have taken a step toward democratizing these advanced tools and making them available to a wide medical audience. The main findings of the study show that 3D models can significantly improve the visualization of complex anatomical structures, facilitate preparation, and increase the accuracy of surgical interventions. The case we used as an example highlights the practical contribution of these models to improving surgical outcomes and reducing operative time.

However, our study also points out some limitations, such as the need for significant technical skills and time resources for model segmentation and optimization, as well as the dependence on the quality of the source images. These limitations emphasize the need for future research to focus on process automation and the improvement of the accuracy and efficiency of 3D models. We believe it provides a solid foundation for how and why such tasks are performed using entirely free software, and it allows interested professionals to create samples for preoperative planning according to their needs. Given that medical personnel have the necessary knowledge to identify relevant anatomical structures but may not necessarily have the engineering expertise to create a printable file, we have chosen a printer with the most user-friendly interface available. We recommend that if not the creation of the 3D object itself, at least the preparation of the printable file should be done in collaboration with specialists in 3D printing, as this is a new, interesting, and promising field of application. Future studies should include a larger number of clinical cases and different anatomical structures to validate and expand the applicability of the methodology. As technology and software advance, we expect these tools to become even more accessible and easy to use, allowing more medical teams to benefit from them and leading to better patient outcomes. This research lays the foundation for the broader adoption of 3D printing in medical practice, emphasizing the importance of interdisciplinary collaboration between medical professionals and engineers to achieve innovation and improve healthcare.
